# Cognitive Outcomes following Transcatheter Aortic Valve Implantation: A Systematic Review

**DOI:** 10.1155/2015/209569

**Published:** 2015-02-15

**Authors:** Ka Sing Paris Lai, Nathan Herrmann, Mahwesh Saleem, Krista L. Lanctôt

**Affiliations:** ^1^Neuropsychopharmacology Research Group, Sunnybrook Health Sciences Centre, Toronto, ON, Canada M4N 3M5; ^2^Faculty of Medicine, University of Toronto, Toronto, ON, Canada M5S 1A8; ^3^Department of Psychiatry, University of Toronto, Toronto, ON, Canada M5T 1R8; ^4^Department of Pharmacology and Toxicology, University of Toronto, Toronto, ON, Canada M5S 1A8

## Abstract

Severe aortic stenosis is the most common valvular heart disease in the elderly in the Western world and contributes to a large proportion of all deaths over the age of 70. Severe aortic stenosis is conventionally treated with surgical aortic valve replacement; however, the less invasive transcatheter aortic valve implantation (TAVI) is suggested for those at high surgical risk. While TAVI has been associated with improved survival and favourable outcomes, there is a higher incidence of cerebral microembolisms in TAVI patients. This finding is of concern given mechanistic links with cognitive decline, a symptom highly prevalent in those with cardiovascular disease. This paper reviews the literature assessing the possible link between TAVI and cognitive changes. Studies to date have shown that global cognition improves or remains unchanged over 3 months following TAVI while individual cognitive domains remain preserved over time. However, the association between TAVI and cognition remains unclear due to methodological limitations. Furthermore, while these studies have largely focused on memory, cognitive impairment in this population may be predominantly of vascular origin. Therefore, cognitive assessment focusing on domains important in vascular cognitive impairment, such as executive dysfunction, may be more helpful in elucidating the association between TAVI and cognition in the long term.

## 1. Introduction

Cardiovascular disease including valvular heart disease contributes to an estimated 36% of all deaths over the age of 70 [1]. In particular, severe aortic stenosis is the most common valvular heart disease in the elderly in the Western world [2] and is associated with reduced quality of life and increased mortality [3, 4]. Conventionally, surgical aortic valve replacement is indicated for patients with severe aortic stenosis. However, transcatheter aortic valve implantation (TAVI) is a less invasive procedure rapidly being adopted as an alternative to surgical aortic valve replacement for those who are inoperable or at high surgical risk [4]. TAVI has been shown to improve survival, with a lower all-cause mortality at one year than surgical aortic valve replacement [4], and is associated with favourable long-term outcomes including improved functional capacity and quality of life [3].

Recent evidence from randomized controlled trials suggests an increased risk of neurological events for up to one year after TAVI in comparison to surgical aortic valve replacement and medical treatment [4, 5]. In particular, diffusion weighted magnetic resonance imaging (DW-MRI) studies have demonstrated that TAVI is associated with a high incidence of cerebral microembolisms [6], a conceivable contributor to cognitive decline. In addition, advanced age and high comorbidity burden, particularly a high prevalence of coronary artery disease, commonly seen in patients undergoing TAVI may contribute to cognitive decline despite intervention. Few studies have assessed cognitive outcomes after TAVI. To date, neurological outcomes research related to TAVI has focused on strokes and transient ischemic attacks. The aim of this paper was to conduct a systematic review of the known information regarding cognitive changes in patients undergoing the TAVI procedure based on studies published to date.

## 2. Methods

Methodology recommended by the PRISMA guidelines for systematic reviews was followed for all analyses [7]. The Cochrane, PsycINFO, Embase, and Medline databases were searched for English-language articles published up to January 2015 assessing cognition before and after TAVI with standardized neuropsychological measures. A sample search strategy (for Embase using OVID) is detailed in Supplementary Table 1 (see Supplementary Materials available online at http://dx.doi.org/10.1155/2015/209569). Studies were included regardless of the route of valve implantation. All studies regardless of the duration of the follow-up period were also included in this review.

## 3. Results

**Figure 1:**
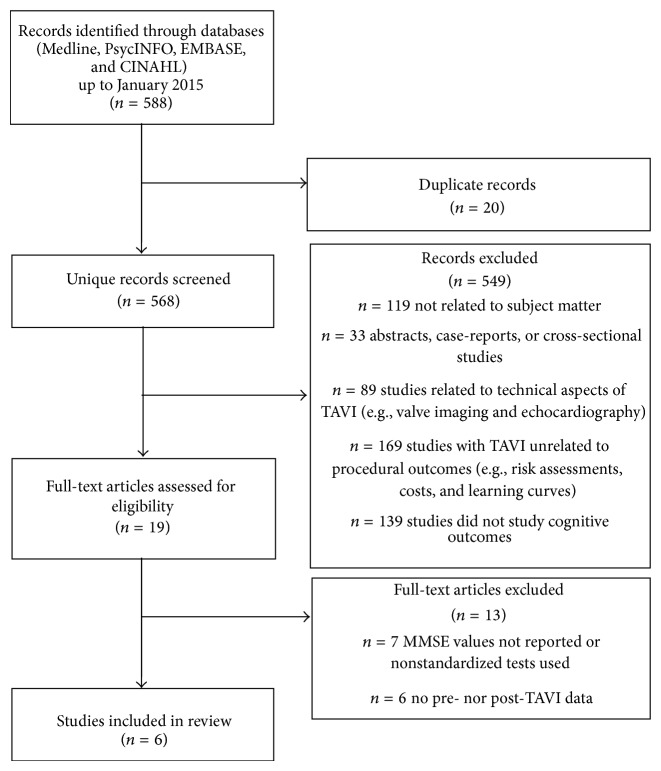
Illustration of search strategy.

Search criteria returned 568 unique records of cognitive assessments in patients undergoing TAVI (Figure 1). Of the total studies, 549 studies were excluded: 119 were not related to the subject matter, 33 studies were abstracts, case-reports, or cross-sectional studies, 89 papers were related to the technical aspects of TAVI (e.g., valve imaging using echocardiography), 169 studies did not look at procedural outcomes (e.g., cost assessments), and 139 studies did not look at cognitive outcomes. Upon review of 19 articles to assess for eligibility for inclusion, 13 studies were excluded: 7 articles did not report scores of the mini-mental status examination (MMSE), a measure of overall cognitive function (global cognition), 1 study did not use standardized tests for cognition, and 6 studies did not report pre- and post-TAVI cognitive test scores. A total of 6 studies that met inclusion criteria were reviewed. All 6 studies assessed cognition before and after TAVI using the MMSE (Table 1). Specific cognitive functions such as attention or verbal memory (specific cognitive domains) were measured in 3 of the 6 studies.

Characteristics of the 349 patients in the eligible studies, including demographics, surgical risk scores, cognitive status, comorbidities, and prevalence of cardiovascular risk factors, are shown in Table 2. The mean age of the populations ranged from 78 to 83 years. Patients undergoing TAVI had variable, albeit high, logistic EuroSCOREs at baseline with mean values that ranged from 15 to 36. At least 56% of the TAVI patients have significant comorbidities such as coronary artery disease, and many have other significant cardiovascular risk factors such as dyslipidemia (77%) and hypertension (93%).

### 3.1. Changes in Global Cognitive Function following TAVI

The MMSE is a widely used screening tool for cognitive impairment where a score equal to or below 24 can be indicative of cognitive impairment [13]. Six prospective cohort studies (Table 1) assessed global cognition following TAVI using the MMSE. Postprocedural assessments took place on average 5 days after the TAVI procedure (with the longest being 10.7 ± 4.9 days) [10] showing no change in cognition [6, 9–12]. However, one study detected a significant increase in MMSE scores as early as 1 month after TAVI [8].

Of the three studies that followed TAVI patients over a period of 3 months, two showed a significant improvement in MMSE scores after TAVI [9, 10] while one did not [12]. However, in the study that did not show improved MMSE scores over 3 months, a significant increase in Montreal Cognitive Assessment (MoCA) score was observed (from 23.4 [95% confidence interval (CI): 22.7–24.0] to 24.3 [95% CI: 23.6–24.9], *P* < 0.001, normal MoCA score ≥ 26) [12]. In all three studies, preserved global cognition was indicated by an average MMSE score ≥26 at follow-up [8–10].

### 3.2. Changes in Specific Cognitive Domains after TAVI

Knipp et al. performed the first study to determine changes in specific cognitive domains in patients undergoing TAVI using a cognitive battery [10]. In that study, verbal and visual short-term memory, working memory, verbal learning, delayed recognition, and verbal fluency were assessed over a span of 3 months after TAVI using the digit span subtest from the Wechsler Memory Scale-Revised, wordlist test, and Regensburg word fluency test. No significant changes in any cognitive domain were found when comparing preoperative test scores to 3-month follow-up scores.

Ghanem et al. used a cognitive battery to assess neuropsychological outcomes over 2 years after TAVI [6]. Using the Repeatable Battery for the Assessment of Neuropsychological Status (RBANS) [14], changes in language, attention, visual and constructional skills, and immediate and delayed memory were measured. While not significant, 4 of the 32 patients remaining in the study at 2-year follow-up showed cognitive decline represented by a decline of one standard deviation in total RBANS score from baseline. A subgroup of 30 patients with a baseline RBANS score of less than 1.5 standard deviations below the population norm was defined as having mild cognitive impairment (MCI). In that group, 6 patients demonstrated incident cognitive decline postprocedurally, with 3 patients maintaining cognitive decline up to the end of the follow-up period. Overall in the MCI subgroup, the RBANS scores for attention, delayed memory, and visual/constructional skills were significantly lower than those with no MCI postprocedurally. The MCI group consistently showed significantly lower scores in these domains up to the 2-year follow-up time-point.

Orvin et al. also assessed cognitive function in patients undergoing TAVI using the quantitative clock drawing test, colors trails tests (CTT1 and CTT2), and Cognistat before and one month after TAVI [8]. Their battery of tests allowed for assessment of attention, language, construction, memory, calculations, reasoning, and executive function. The authors found a significant improvement in the Cognistat scores between before and after TAVI. There were no significant differences in the CTTs or the clock drawing test, although 24 of 36 patients had better scores on the clock drawing test at follow-up.

## 4. Discussion

Studies assessing cognition following TAVI have generally found cognitive improvement or preservation. Specifically, global cognition significantly improved or remained unchanged while changes in individual cognitive domains remained unchanged for up to 2 years following TAVI. These findings are consistent with recent meta-analyses, which found improvements in processing speed, executive function, verbal short-term memory, and working memory up to 1 year after coronary artery bypass graft surgery [15, 16].

Improvements in cognition after cardiovascular surgery such as TAVI may be attributed to significant changes in hemodynamic parameters, particularly cardiac output [17]. Cardiac output is integral for meeting the metabolic demands of the brain [18]. However, insufficient cardiac output commonly seen in patients with severe aortic stenosis may lead to cerebral hypoperfusion, a primary contributor to cognitive decline [19]. Low cardiac output due to severe cardiovascular disease has been shown to be associated with a faster rate of cognitive decline in the attention, executive, and psychomotor domains [20, 21]. It has been shown that impaired cerebral blood flow may be reversible [22] and that restoration of cerebral blood flow can subsequently lead to improved cognitive function [23]. Since cardiac output has been shown to improve after TAVI [17], cognitive preservation or improvement after TAVI is concordant with the hypothesis that increased cardiac output and improved cerebral blood may be important contributors to cognitive preservation or improvement.

Alleviation of physical symptoms and subsequent improvement in functional status may also contribute to improvements in cognition after TAVI. It is known that patients with severe aortic stenosis experience a high degree of fatigue due to reduced ejection fraction [24]. Because left ventricular function and ejection fraction significantly improve after TAVI [24], fatigue is likely reduced along with functional recovery. Therefore, patients may also be performing better on cognitive testing due to improvement in functional symptoms and reduced fatigue after TAVI.

Despite the favourable hemodynamic improvements following TAVI, it is important to note that patients with severe aortic stenosis are at an increased risk of cognitive impairment due to advanced age and high comorbidity burden. More specifically, patients with severe aortic stenosis have a high prevalence of coronary artery disease and typically present with multiple vascular risk factors (Table 2) both of which are known to be associated with an increased risk of vascular cognitive impairment [25]. Key features of vascular cognitive impairment include subcortical deficits involving executive function and processing speed [26]. Findings indicating the frontal lobe as the region with the highest number of microembolic lesions [10] after TAVI and deficits in delayed memory recall and visual/constructional skills [6] domains known to be dependent on cortical processing are consistent with the hypothesis that vascular cognitive impairment may be more prevalent in this population. Use of a neuropsychological battery well validated for the study of vascular cognitive impairment such as the one recommended by National Institute of Neurological Disorders and Stroke (NINDS) and the Canadian Stroke Network (CSN) (Brief Visuospatial Memory Test-Revised, Hopkins Verbal Learning Test-Revised, Controlled Oral Word Association Test, animal fluency, Trail Making Test Parts A and B, and Symbol Digit Modalities Test) [27] may be more appropriate for studying cognitive changes in patients with a high prevalence of cardiovascular comorbidities, such as those undergoing TAVI.

All 6 studies measured global cognitive function before and after TAVI using the MMSE as a measure of cognitive decline. It is of interest to note that studies reported significant increase in MMSE scores following TAVI even though more sensitive and specific measures of cognition did not appear to change. It is possible that improvements in MMSE scores may be due to learning effects [28]; however, the test-retest reliability of the MMSE ranged from 0.83 to 0.95 for an interval as short as 1 day across various populations [29]. In addition, while the scores improved, a 1-point change in MMSE scores may not be clinically relevant; wide standard deviations in MMSE scores in the included studies consistent with findings in Alzheimer's Disease Neuroimaging Initiative cohort [30] and other reports [31, 32] suggest that differences between clinical changes and possible effects of confounding variables such as sex, education [32], and functional status [31] may be difficult to interpret. The MMSE has also been suggested to be insufficient in probing executive function and may lack sensitivity in detecting subtle memory impairment [27]. Therefore, despite the widely accepted use of the MMSE as a screening tool for cognitive impairment, more reliable measures such as the MoCA, also recommended by the NINDS-CSN criteria, may be more sensitive and superior to the MMSE in measuring global cognitive impairment in those that have undergone TAVI.

Studies assessing cognitive changes in specific domains following TAVI may also not have effectively assessed vascular cognitive impairment, particularly executive function. In the study by Ghanem et al. [6], mild cognitive impairment was assessed using the RBANS, which may lack clinical utility in this population. When compared to a battery consisting of tests recommended by the NINDS-CSN criteria, it was found that while the RBANS had good specificity, it lacked sensitivity to detect cognitive impairment when a cut-off of one standard deviation below the norm was applied (sensitivity = 55% using the total RBANS score) [33]. Orvin et al. study was the only one to specifically assess executive function deficits [8] which found preserved executive function in TAVI patients after 1 month. However, the NINDS-CSN criteria suggest that category and letter fluency, category cueing, processing speed, activation, and set shifting need to be sampled in order to provide a complete assessment of frontal lobe function.

Mechanisms underlying cognitive changes after TAVI are unclear. It was hypothesized that a high incidence of cerebral microembolisms associated with both vascular dementia and Alzheimer's disease [34] may be an important contributor to cognitive decline in this population. It has been suggested that the higher incidence of embolisms associated with TAVI is likely due to procedural factors [9, 11, 12] including procedural route and valve type. Of the two procedural approaches, the transfemoral approach involves manipulation of the ascending aorta and the aortic arch and potentially dislodgement of particles into the bloodstream compared to transapical TAVI and is therefore associated with an increased risk of cerebrovascular events [11]. Valve type may also lead to an increased number of cerebral microembolisms; the Edwards-SAPIEN (ES) valve is rapidly implanted increasing scraping of calcified debris from the aortic valve. The Medtronic CoreValve (MCV) valve on the other hand involves slow, stepwise opening of the aortic valve which would be less likely to dislodge calcified debris.

To date, there have been 4 studies that have performed DW-MRI to detect microembolic lesions and concurrently measured cognitive function [6, 10–12] (Table 3). It was observed that the highest frequency of cerebral microembolisms was detected in the frontal lobe; however, executive function was not specifically assessed. Despite diffuse microembolisms observed in both hemispheres, no decline was noted within any cognitive domain [10]. Comparing individuals who had cerebral microembolisms with those who had no embolisms postprocedurally, there was no significant difference within any domain [6]. When comparing procedural routes, significant increases in MMSE scores in the transfemoral TAVI patients were observed at 3-month follow-up compared to transapical TAVI patients in a study done by Kahlert et al. [9]. However, the transfemoral TAVI patient group started with a higher baseline MMSE score indicating a possible “ceiling effect” in transfemoral TAVI group. In addition, while the earlier study by Kahlert et al. found no differences in MMSE scores between the ES and MCV patient groups [12] at follow-up, their later study found that those with the ES valve showed a significant increase in MMSE scores over 3 months compared to those with the MCV valve [9] even though the number of cerebral microembolisms was higher in those with the ES valve [9] (Table 3). A ceiling effect may have also influenced these results since the MCV valve patient group had a higher baseline MMSE score and therefore had less potential for change. These findings suggest that microembolic lesions may not be clinically significantly consistent with recent indications that periprocedural microembolic lesions may be silent [12, 35]. Conversely, recent evidence suggests that because many of these lesions also occur within the cerebellar area, they may be important in loss of coordination and functional deficits in patients after TAVI [12].

This review was limited by a heterogeneous patient population as indicated by wide variability in logistic EuroSCOREs at baseline. Follow-up times varying in length from 3 days to 3 months and different cognitive assessments used made it difficult to compare data. Furthermore, cerebrovascular disease was reported inconsistently and neither carotid nor cerebrovascular disease was accounted for in the analyses. Practice effects may have contributed to the lack of change or improvement in cognition. The lack of control groups, short duration of follow-up, and small sample sizes also make it difficult to make concrete conclusions.

## 5. Conclusions

TAVI is becoming an increasingly popular alternative to surgical aortic valve replacement as the procedure improves survival, is less invasive, and can be performed in high-risk patients with severe aortic stenosis. Studies have generally shown no change or cognitive improvement after TAVI possibly due to beneficial improvements in hemodynamic status. Cognitive preservation or improvement after TAVI in a population with a declining cognitive trajectory already set in motion by aging and significant underlying vascular disease may suggest benefit. The balance between positive changes associated with TAVI and already existing predisposing factors is currently obscured by various limitations in these studies. Larger prospective longitudinal studies are needed to validate current findings using a more comprehensive battery to assess vascular cognitive impairment in order to clearly elucidate the association between TAVI and cognition.

## Supplementary Material

Supplementary Table 1: Sample search strategy (OVID searching EMBASE).
This appendix details the search strategy used to identify English-language articles published up to January 2015 assessing cognition before and after TAVI with standardized neuropsychological measures. Databases searched for included Cochrane, PsycINFO, Embase, and Medline. An example search strategy for Embase using OVID is detailed below. Steps 1 through 6 were used to search for TAVI studies, steps 7 through 18 were used search for studies that have studied cognition or neuroimaging, step 19 was used to search for TAVI studies that have looked at cognition or neuroimaging, and step 20 was used to include only English language articles studying humans published from 1990 to the date of search.

## Figures and Tables

**Table 1:** Summary of studies measuring global cognition after TAVI using the MMSE.

Study	*n*	Type of TAVI	Baseline	After procedure	1-month F/U	3-month F/U
Orvin et al., 2014 [8]	36	TF (MCV) = 31 TA (MCV) = 5	25.9 ± 3.3	—	27.6 ± 2.4 *P* < 0.001	—

Ghanem et al., 2013 [6]	111	TF (MCV) = 95 TF (ES) = 16	25.4 ± 3.4	25.4 ± 3.3^a^ 25.1 ± 3.8^b^ *P* = 0.58	—	—

Kahlert et al., 2012 [9]	83	*Overall *	27.9 (27.5–28.3)	27.7 (27.3–28.2) *P* = 0.521	—	28.3 (28.0–28.7) *P* < 0.001
		*TF (MCV) = 32 *	*28.0* *(27.5–28.5) *	*27.7* *(27.3–28.2)* *P* = 0.488	—	*28.4* *(27.9–28.9)* *P* = 0.083
		*TF (ES) = 26 *	*27.3* *(26.1–28.6) *	*27.6* *(26.4–28.7)* *P* = 0.400	—	*28.1* *(27.0–29.1)* *P* = 0.003
		*TA (ES) = 25 *	*28.3* *(27.8–28.8) *	*28.0* *(27.4–28.5)* *P* = 0.256	—	*28.6* *(28.1–29.0)* *P* = 0.096

Knipp et al., 2013 [10]	27	TA (ES) = 27		Δ−0.72 ± 1.42^c^ *P* > 0.05	—	Δ0.95 ± 1.20^c^ *P* < 0.05

Rodés-Cabau et al., 2011 [11]	60	TF (ES) = 29 TA (ES) = 31	28 (17–30)	28 (16–30) *P* = 0.14	—	—

Kahlert et al., 2010 [12]	32	TF (MCV) = 10	28.9 (28.2–29.6)	28.0 (27.0–29.0) *P* > 0.05	—	28.9 (28.0–29.0) *P* > 0.05
		TF (ES) = 22	28.1 (26.7–29.5)	28.3 (27.1–29.6) *P* > 0.05		28.1 (26.7–29.7) *P* > 0.05

*n* = number, TAVI = transcatheter aortic valve implantation, MMSE = mini-mental status examination, TF = transfemoral, TA = transapical or transaxillary, ES = Edwards-SAPIEN, MCV = Medtronic CoreValve, and F/U = follow-up.

All studies were observational prospective cohort study. *P* values are for MMSE scores from the specified time-point compared to baseline unless otherwise stated. Normal score for MMSE is 24 or above.

^a^Subgroup with no risk of cognitive decline.

^b^Subgroup with risk of cognitive decline.

^c^Change in *z*-score after procedure to follow-up.

**Table 2:** Summary of TAVI patient population characteristics at baseline in current studies.

Parameter	Orvin et al., 2014 [8]	Ghanem et al., 2013 [6]	Kahlert et al., 2012 [9]	Knipp et al., 2013 [10]	Rodés-Cabau et al., 2011 [11]	Kahlert et al., 2010 [12]	
*n*	36	111	83	27	60	22 (ES)	10 (MCV)

Demographics							
Age, mean ± SD	82.2 ± 4.2	80 ± 6	80.6 (79.3–81.8)	82.2 ± 4.7	83 ± 7	78.3 (76.4–80.2)	83.8 (79.2–88.4)
Male gender, % (*n*)	52.8 (19)	54 (60)	57.8% (35)	74.1 (20)	50 (30)	36 (14)	60 (4)

Risk scores							
Logistic EuroSCORE, mean ± SD	14.9 ± 11.4	24.3 ± 14.7	20.7 (17.8–23.5)	36.4 ± 13.2	18.9 ± 12.8	22.8 (16.5–29.2)	17.9 (12.0–23.7)
Society of Thoracic Surgeons Score	7.4 ± 4.1	8.5 ± 5.4	6.7 (5.7–7.7)	—	7.7 ± 4.6	—	—

Cognitive status							
Mini-mental status exam, mean ± SD	25.9 ± 3.3	25.4 ± 3.4	27.9 (27.5–28.3)	—	28 (17–30)	28.9 (28.2–29.6)	28.1 (26.7–29.5)
Mild cognitive impairment, % (*n*)	—	27 (30)	—	—	—	—	—

Comorbidities							
Coronary artery disease, % (*n*)	—	63 (71)	55.4 (46)	55.5 (15)	73 (44)	64 (14)	50 (5)
Renal dysfunction, % (*n*)	30.5 (11)	—	16.9 (14)	—	88 (53)^a^	32 (7)	10 (1)

Cardiovascular risk factors							
Obesity, % (*n*)	—	—	50.6 (42)	—	88 (53)	41 (9)	0 (0)
Smoking, % (*n*)	—	18 (20)	21.7 (18)	29.6 (8)	2 (1)	23 (5)	20 (2)
Diabetes, % (*n*)	30.5 (11)	31 (35)	30.1 (25)	29.6 (8)	25 (15)	27 (6)	40 (4)
Hypertension, % (*n*)	88.9 (32)	98 (109)	97.6 (81)	100 (27)	75 (45)	95 (21)	90 (9)
Dyslipidemia, % (*n*)	83.3 (30)	81 (90)	72.3 (60)	74.1 (20)	73 (44)	86 (19)	50 (5)

*n* = number, TAVI = transcatheter aortic valve implantation, SD = standard deviation, ES = Edwards-SAPIEN, MCV = Medtronic CoreValve.

^a^Renal dysfunction indicated by estimated glomerular filtration rate <60 mL/min.

**Table 3:** Summary of studies utilizing DW-MRI to view cerebral ischemia and assessed global cognition using the MMSE.

Study	*n*	Type of TAVI	Time after TAVI that DW-MRI was performed	Number of patients with lesions	Incidence of patients with lesions	Total number of lesions	Average number of lesions per patient
Ghanem et al., 2013 [6]	111	TF (MCV) = 95 TF (ES) = 16	3 days	36^a^	64%	—	—

Knipp et al., 2013 [10]	27	TA (ES) = 27	10.7 ± 4.9 days	12	58%	22	1.83

Rodés-Cabau et al., 2011 [11]	60	TF (ES) = 29 TA (ES) = 31	4 ± 1 days	19 22	66% 71%	83 168	4.37 7.63

Kahlert et al., 2010 [12]	32	TF (MCV) = 10 TF (ES) = 22	3.4 days (2.5–4.4)	8 19	80% 86%	26 89	3.25 4.68

*n* = number, TAVI = transcatheter aortic valve implantation, MMSE = mini-mental status examination, DW-MRI = diffusion weighted magnetic resonance imaging, ES = Edwards-SAPIEN, and MCV = Medtronic CoreValve.

^a^Only 56 patients were able to undergo postprocedural imaging.
