# Supplementary material for: Cognitive Outcomes following Transcatheter Aortic Valve Implantation: A Systematic Review
Source: Cardiovasc Psychiatry Neurol. 2015 Feb 15;2015:209569. doi: 10.1155/2015/209569 (PMC4345202; doi:10.1155/2015/209569)
Supplement: Supplementary file 1 — Supplementary Table 1: Sample search strategy (OVID searching EMBASE). This appendix details the search strategy used to identify English-language articles published up to January 2015 assessing cognition before and after TAVI with standardized neuropsychological measures. Databases searched for included Cochrane, PsycINFO, Embase, and Medline. An example search strategy for Embase using OVID is detailed below. Steps 1 through 6 were used to search for TAVI studies, steps 7 through 18 were used search for studies that have studied cognition or neuroimaging, step 19 was used to search for TAVI studies that have looked at cognition or neuroimaging, and step 20 was used to include only English language articles studying humans published from 1990 to the date of search. [file 209569.f1.pdf]

APPENDIX

Supplementary Table 1: Sample search strategy (OVID searching EMBASE)

#	**Searches**
1	(transcatheter aortic valve implantation or transcatheter aortic valve replacement).mp.
2	exp transcatheter aortic valve implantation/
3	exp heart valve prosthesis/
4	(TAVI or TAVR or transcatheter).mp.
5	exp heart catheterization/
6	1 or ((2 or 3) and (4 or 5))
7	exp cognition/
8	exp cognitive defect/
9	((cognitive or cognition or brain or memory or processing) adj3 (impair* or disorder or change or compromise or decline or speed or test)).mp.
10	exp memory/
11	exp memory disorder/
12	exp neuropsychological test/
13	exp neuroimaging/

14	exp diffusion weighted imaging/
15	diffusion weighted mri.mp.
16	exp brain embolism/
17	cerebral embolism.mp.
18	or/7-17
19	6 and 18
20	limit 19 to (human and english language and yr="1990 -Current")
